# Molecular-Assisted Pollen Grain Analysis Reveals Spatiotemporal Origin of Long-Distance Migrants of a Noctuid Moth

**DOI:** 10.3390/ijms19020567

**Published:** 2018-02-13

**Authors:** Hong Chang, Jianglong Guo, Xiaowei Fu, Yongqiang Liu, Kris A. G. Wyckhuys, Youming Hou, Kongming Wu

**Affiliations:** 1 State Key Laboratory of Ecological Pest Control for Fujian and Taiwan Crops and Fujian Province Key Laboratory of Insect Ecology, Fujian Agriculture and Forestry University, Fuzhou 350002, China; Hong.Chang.PhD@gmail.com; 2 State Key Laboratory for Biology of Plant Diseases and Insect Pests, Institute of Plant Protection, Chinese Academy of Agricultural Sciences, Beijing 100193, China; lyq364467268@163.com (Y.L.); kagwyckhuys@gmail.com (K.A.G.W.); 3 College of Plant Protection, Shenyang Agricultural University, Shenyang 110866, China; jianglongguo88@163.com; 4 Department of Plant Protection, Henan Institute of Science and Technology, Xinxiang 453003, China; fxw1983@126.com

**Keywords:** plant-pollinator, *Agrotis segetum*, pollen grain analysis, DNA barcoding, migration, aerobiology

## Abstract

Pollen grains are regularly used as markers to determine an insect’s movement patterns or host (plant) feeding behavior, yet conventional morphology-based pollen grain analysis (or palynology) encounters a number of important limitations. In the present study, we combine conventional analytical approaches with DNA meta-barcoding to identify pollen grains attached to migrating adults of the turnip moth, *Agrotis segetum* (Lepidoptera: Noctuidae) in Northeast China. More specifically, pollen grains were dislodged from 2566 *A. segetum* long-distance migrants captured on Beihuang Island (Bohai Sea) and identified to many (plant) species level. Pollen belonged to 26 families of plants, including Fagaceae, Oleaceae, Leguminosae, Asteraceae, Pinaceae and Rosaceae, including common species such as *Citrus sinensis*, *Olea europaea*, *Ligustrum lucidum*, *Robinia pseudoacacia*, *Castanopsis echinocarpa*, *Melia azedarach* and *Castanea henryi*. As the above plants are indigenous to southern climes, we deduce that *A. segetum* forage on plants in those locales prior to engaging in northward spring migration. Our work validates the use of DNA-assisted approaches in lepidopteran pollination ecology research and provides unique and valuable information on the adult feeding range and geographical origin of *A. segetum*. Our findings also enable targeted (area-wide) pest management interventions or guide the future isolation of volatile attractants.

## 1. Introduction

Plant-pollinator interactions can reveal co-evolutionary processes in both animal and plant communities [1]. There are a variety of pollination modes in nature and entomophily has been one of the determinants of the ecological and evolutionary success of angiosperms and the associated coevolution with multiple orders of insects over the past 100 million years [2]. Scientific research has tried to shed light upon these plant-pollinator interactions and has investigated both nectar and pollen feeding by adult herbivores [3,4,5]. Pollen-grain analysis (or palynology) is one approach that is regularly used to study the role of insects in pollination [5,6,7,8,9].

Aside from revealing plant-pollinator linkages, pollen can equally serve as a natural marking material to determine an insect’s host plant range and its related geographical origin or movement patterns in time or space [5,9]. Pollen identification is particularly useful to study insect migration for four reasons: (a) entomophily-dependent plant species have evolved pollen that adheres readily to the insect body [5]; (b) the rigid exterior (or exine) of pollen grains is composed of sporopollenin, one of the most enduring natural polymers [10]; (c) pollen grains are distinctive and can be used to identify genus of originating plants [11]; (d) the distribution and flowering periods of most plants are well known, which helps to establish both temporal as spatial facets related to origin of captured insects [5].

Pollen identification is done with the aid of a light microscope (LM) or scanning electron microscope (SEM) and guided by pollen identification keys, micrographs and reference collections. Though LM-based pollen identification is constrained by its low level of resolution, the drawback to using SEM for pollen analyses is the regular absence of reference micrographs and its increased cost and time requirements [3,12]. Furthermore, though pollen grains are morphologically distinct, microscopy-based approaches regularly do not permit separating pollen grains beyond the plant genus level [13,14]. The recent development of DNA meta-barcoding offers unprecedented opportunities to advance palynology-based ecological research on a number of frontiers [15,16,17,18]. DNA-based identification of pollen grains has become prominent in ecological studies, as a viable alternative or complement to conventional approaches. In recent years, this method has been successfully used to determine honeybee foraging preferences and the floral composition of honey [19,20], or to shed light upon pollen foraging behavior of flower-visiting insects [21].

Molecular-guided palynology equally carries promise to illuminate aspects of the ecology of Lepidoptera. Although most lepidopteran species are herbivorous during the larval stage [22], their adults visit flowers to feed on nectar and/or pollen [23]. As such, Lepidoptera are one of the most important groups of pollinators and use floral nectar as their principal carbohydrate source. While only a few species within the genera *Heliconius* and *Laparus* have evolved adaptations to pollen-feeding [24], the majority of species benefit from pollen-derived nutrients when consuming pollen-contaminated nectar [25,26]. Also, most lepidopterans are exposed to pollen as it is attached to the proboscis or other body parts during flower visiting. Hence, palynology can reveal migration patterns of Lepidoptera, as exemplified by the pollen-guided elucidation of the migration origin of *Helicoverpa zea* in Arkansas [5], *Agrotis ipsilon* and *Pseudaletia unipuncta* in Iowa and Missouri [27] and *H. zea* and *Trichoplusia ni* collected in Oklahoma [28]. In the present study, metabarcoding was used along with pollen morphology to identify the pollen species.

The turnip moth, *Agrotis segetum* Denis and Schiffermaller (Lepidoptera: Noctuidae), is a pest of wide geographic distribution and agricultural importance [29,30,31]. Previous research has shown that Chinese populations of *A. segetum* undertake seasonal migration [32]. However, the exact geographic origin of moths and their migration routes has still not been confirmed. In this study, we concurrently employ morphologically-based approaches and DNA meta-barcoding to illuminate the host relationship and geographic origin of *A. segetum* moths. Our work validates the use of DNA-assisted palynology in aerobiology and lepidopteran ecological research and has important implications for future (area-wide) pest management of *A. segetum* and other migratory lepidopteran pests.

## 2. Results

### 2.1. Plant Hosts Inferred from Pollen

**Figure 1:**
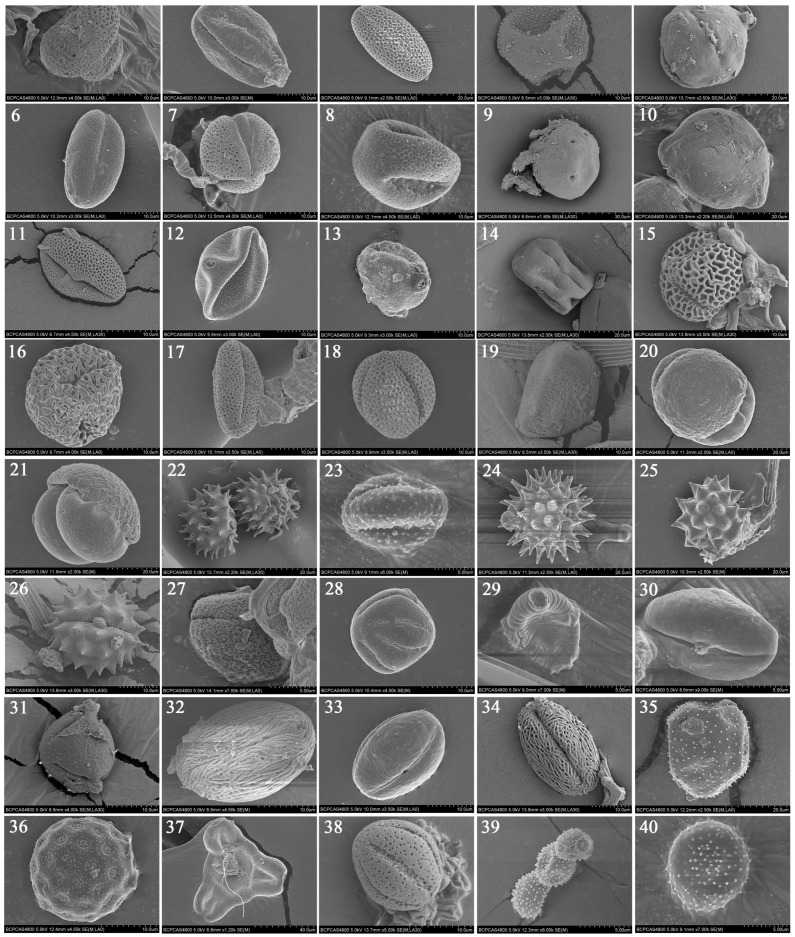
Scanning Electron Microscopy (SEM) microphotographs of the examined pollen species: 1. *Citrus sinensis*; 2. *Heliotropium* L.; 3. Liliaceae; 4. Cupressaceae; 5. *Robinia pseudoacacia*; 6. Leguminosae; 7. *Amorpha fruticosa*; 8. *Cercidium* L.; 9. *Pterocarya rhoifolia*; 10. *Elaeagnus umbellate*; 11. *Fendlera* Engelm. & Gray; 12. *Corylus* L.; 13. *Betula* L.; 14. *Melia azedarach*; 15. *Olea europaea*; 16. *Ligustrum lucidum*; 17. Brassicaceae; 18. *Brassica* L.; 19. Alliaceae; 20. *Pinus* L.; 21. Pinaceae; 22. *Helianthus* L.; 23. *Artemisia* L.; 24. Asteraceae; 25. *Gnaphalium* L.; 26. *Chrysanthemum* L. 27. *Dendromecon* Benth.; 28. *Eschscholtzia* Cham.; 29. *Polygala* L.; 30. *Castanea henryi*; 31. *Castanopsis echinocarpa*; 32. *Ailanthus Desf.*; 33. Rosaceae; 34. Rosaceae; 35. *Adenophora trachelioides*; 36. *Chenopodium album*; 37. *Gaura* L.; 38. *Galium* L.; 39. *Pilea Lindl.*, *nom. conserv*.; 40. Lauraceae. The scale bar has been showed on the bottom of each photograph: 1, 2, 4, 6–8, 11–13, 15–19, 26, 28, 31–34, 36, 38: 10 μm; 3, 5, 10, 14, 20–22, 24, 25, 35: 20 μm; 9: 30 μm; 23, 27, 29, 30, 39, 40: 5 μm; 37: 40 μm.

Over the course of the study, we collected a respective total of 1380 and 1186 male and female adult *A. segetum*, of which 17.03% had adhering pollen grains on the proboscis. For the majority of individuals which adhered pollen by body (i.e., 93.4%), pollen grains of one single plant species adhered to the adult body, while the remainder of individuals harbored grains of multiple species. We recorded a total of 40 pollen grains from at least 26 families. Using a combination of DNA sequences and pollen morphology, 12 of the 40 samples were identified to species level: *Castanea mollissima* Blume, *Pterocarya rhoifolia* Siebold et Zucc., *Olea europaea* L., *Amorpha fruticosa* Linn., *Ligustrum lucidum* Ait., *Robinia pseudoacacia* L., *Castanopsis echinocarpa* Miq., *Citrus sinensis* Blanco, *Melia azedarach* L., *Elaeagnus umbellata* Thunb., *Chenopodium album* L. and *Adenophora trachelioides* Maxim (Table 1, Figure 1, Supplementary Text S1) and 16 of the samples to the genus level, including *Pinus* L., *Heliotropium* L., *Corylus* L., *Betula* L., *Ailanthus* Desf., *Brassica* L., *Cercidium* L., *Artemisia* L., *Pilea* Lindl., nom. conserv., *Gnaphalium* L., *Polygala* L., *Galium* L., *Gaura* L., *Fendlera* L., *Chrysanthemum* L. and *Helianthus* L. The remainder of samples were identified according to pollen morphology and the flowering periods of the pollen plants, thus permitting identification at the genus (*Dendromecon* Benth., *Eschscholtzia* Cham.) or family level (Asteraceae, Pinaceae, Brassicaceae, Leguminosae, Rosaceae, Liliaceae, Cupressaceae, Alliaceae and Lauraceae). The respective success rates for species-level and genus-level identification using a combination of pollen morphology, DNA meta-barcoding and distribution data were 30% and 70%. The exclusive use of DNA barcoding had a respective success rate of 12.5% and 70% and the reliance upon pollen morphology yielded correct results in a respective 15% and 75% of cases.

### 2.2. Annual and Seasonal Differences in Pollen Adherence Ratio

From the 2566 *A. segetum* adults that were captured and analyzed for pollen grains, variable annual percentages of pollen-carrying individuals were recorded (Table 2). The level of pollen-grain adherence among *A. segetum* adults significantly differed between years (*χ*^2^ = 111.874, *df* = 3, *p* < 0.0001). Overall, 437 pollen grains representing 26 families, 18 genera and 12 species were found in the samples (Table 2).

The relative percentage of adult (male, female) *A. segetum* retaining pollen showed important inter-annual variation, with the highest level of pollen grain adherence recorded in 2015. Overall, there were no significant sex-related differences in the frequency of pollen grain adherence on *A. segetum* adults, either for years groups (2014–2017) (Table 3), with 25.26% of female and 27.23% of male moths contaminated with plant pollens, or years analyzed individually: 2014, 2015, 2016 and 2017 (Table 3).

**Figure 2:**
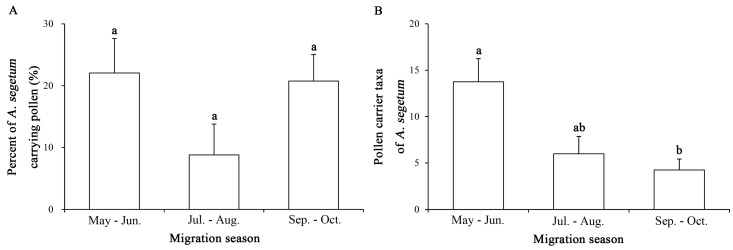
(**A**) Frequencies of pollen grain adherence on migratory *A. segetum* adults, as sampled in Beihuang Island (Bohai Sea) during different stages of the migration season in 2014–2017; (**B**) Number of taxa of pollen adhering to migratory *A. segetum* adults in different stages of the migration season. Bars sharing the same letter reflect absence of statistically significant differences (*p* > 0.05, Tukey’s HSD test).

Furthermore, the frequency of pollen grain adherence in *A. segetum* migrant individuals did not differ among successive stages of the year-long migration season (*F*_2,9_ = 2.843, *p* = 0.11). Meanwhile, the number of pollen taxa obtained from *A. segetum* during May and June was significantly higher than during other stages of the migration season (*F*_2,9_ = 7.246, *p* < 0.013) (Figure 2).

### 2.3. Intra-Annual Shifts in Pollen Taxa

Over the course of a given year, there were important seasonal differences in the type of pollen taxa adhering to migrant *A. segetum*. In early-season, pollen grains from Pinaceae (31.9%), Leguminosae (10.34%) and Oleaceae (9.48%) were most commonly recorded (Table 4). This shifted in the middle period to Brassicaceae (31.33%), Chenopodiaceae (16.87%) and Onagraceae (16.87%) and finally to Asteraceae (68.85%), Urticaceae (14.75%) and Chenopodiaceae (9.02%) in the late period. Collectively, pollen grains of Asteraceae (20.82%), Pinaceae (19.45%) and Brassicaceae (10.53%) were encountered more frequently than from any other family.

### 2.4. Characteristics of Pollen-Bearing Host Plants

**Figure 3:**
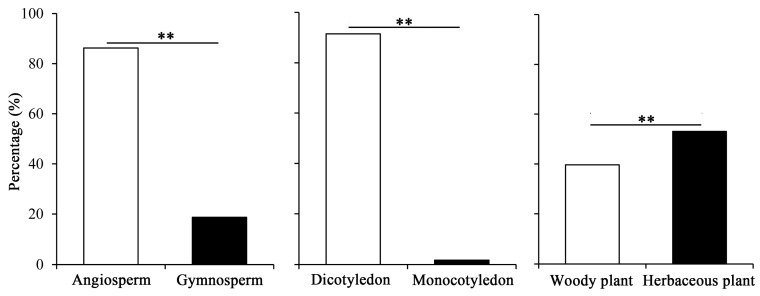
Characteristics of host plants, associated with pollen grains obtained from *A. segetum* migrant individuals during 2014–2017. Double asterisks (**) indicate a statistically significant difference (*p* < 0.01, *Chi-squared* test).

Pollen grains that were collected from migrant *A. segetum* populations originated from a broad range of plant species, including herbs, shrubs and vines. Overall, Angiosperm and Dicotyledonous plants were more common than Gymnosperms (*χ*^2^ = 157.56, *df* = 1, *p* < 0.001) or Monocotyledons (*χ*^2^ = 327.41, *df* = 1, *p* < 0.001). Also, the proportion of herbaceous plants was significantly higher than woody plants (*χ*^2^ = 7.401, *df* = 1, *p* < 0.007) (Figure 3).

## 3. Discussion

Pollen grains have been commonly used as a natural marker to reveal foraging patterns or host plant associations for different types of pollinators [3,4,24]. In this study, we employed conventional morphology-based pollen identification, DNA meta-barcoding and published information on the geographical distribution of plants to reveal the host plant foraging range of Chinese populations of the turnip moth, *A. segetum*.

More specifically, we showed that migrating *A. segetum* adults carry pollen grains from 24 families within the angiosperms and two different gymnosperm families. This is in line with other records of angiosperm plants as common (adult feeding) host plants for Noctuid moths [27,28,33,34]. And *A. segetum* adults may also visit gymnosperm plants and eat their pollen and/or nectar. This result was similar to that for *A. ipsilon* [33]. Meanwhile, no sex-related differences were recorded in the frequency of pollen adherence, as equally observed for *A. ipsilon* [33] and *Mythimna separate* [34]. This finding does differ from *Heliconius ethilla*, in which females consistently carry the largest pollen load [24]. Such species-specific differences can be ascribed to particular courtship behavior, in which resources as accumulated by male moths can be passed onto females during mating [35], however, female *Heliconius* populations would not require that a male’s due to daily spermatophore production, so females carry the largest pollen load than that of males [24]. Our results indicate that *A. segetum* adults visit herbaceous plants more often than woody plants, which differs from *A. ipsilon* [33] and *M. separate* [34]. Different insects may have the different preference of host plants and different host plants can play the important roles in the population increase of insects [36,37]. Pollen from a particular plant species can also be ingested by foraging adults and impact on adult survival and reproduction in many species, such as *H. zea*, *S. exigua*. [38,39]. For species that exclusively feed on floral resources, the type of host plants and their spatial distributions can shape their (meta-) population structure and also affect their temporal dynamics [39]. Hence, further research is needed to assess *A. segetum* preference for certain host plants and their relative contribution to population dynamics.

Aside from revealing plant-pollinator linkages, palynology can also help determine an insect’s migration routes, geographical origin, habitat association and the diversity of (adult) food sources [3,12,40]. Pollen-grain analysis has revealed migration patterns of multiple Lepidoptera species [5,27,41,42]. For example, *H. zea*, *A. ipsilon* and *P. unipuncta* adults in northern parts of the United States carried exotic pollens from Texas, suggestive of a migration range that surpasses several hundred km [5,27]. Previous work has shown that *A. segetum* is equally a long-distance migratory pest [32]. Our detection of pollen from plants that occur in southern China (e.g., *C. sinensis*, *M. azedarach*, *O. europaea*, *L. lucidum*, *R. pseudoacacia*, *C. echinocarpa* and *C. henryi*) revealed how *A. segetum* adults captured in Beihuang island could have migrated over distances >800 km [43]. Early-season migrants thus likely foraged on these flowering plants and subsequently engaged in northward spring migration.

Meanwhile, the above could equally explain why spring-collected moths had significantly more adhering pollen grains as compared to summer or autumn migrants. Similar patterns were observed for *H. zea* and other noctuid moths [27,28,33,34]. Furthermore, other factors that may relate to the higher frequency of pollen grain adherence in spring populations are the increased availability of attractive plants; flowering phenology and time of nectar-production in particular plant species; relative availability of alternate food sources such as honeydew, floral nectar or plant exudates; moth age and associated habitat preferences; intensity and frequency of proboscis-cleaning after feeding; and the relative abundance of flowering plants on which moths are not exposed to pollen [27,28]. However, as a general trend, more plant species are available as food sources for noctuids in the spring as compared with summer and fall seasons.

The relative importance of a particular (plant) taxon as a foraging resource can be inferred through the relative frequency of its adhering pollen grains on field-caught individuals [44]. Our work revealed significant seasonal differences in the pollen taxa adhering to *A. segetum* migrant individuals, with increased importance of Pinaceae, Brassicaceae and Asteraceae during early-, mid- and late-season, respectively. These temporal shifts in the relative importance of particular plant taxa for *A. segetum* populations can be related to climatic conditions and geographic location influencing presence of particular plants and species-specific flowering or pollen-shedding regimes. While the greatest diversity of flowering plants occurs during the spring and most Rosaceae bloom during this season, flowering Asteraceae are relatively more important during the fall. Though Leguminosae, Brassicaceae and Asteraceae had been identified as primary foraging resources for *A. segetum* [45,46], our work clearly shows the importance of a far broader range of plant species. Also, as there are important differences between externally-attached pollen grains and those ingested by foraging noctuid adults [9], our work might even underestimate the foraging range of *A. segetum*.

Nectar-feeding moths can be attracted by the odors of their floral hosts [47,48]. Floral volatiles play an important role in plant-insect communication [49]. Host plant volatiles have been proposed as a potential lure for insects and as a means to monitor and forecast populations of insects [50,51]. We showed that *A. segetum* moths were effective pollinators of *C. sinensis*, *M. azedarach*, *O. europaea*, *L. lucidum*, *R. pseudoacacia*, *C. echinocarpa*, *C. henryi* and other plants. The flowers of these plants may contain specific attractant volatile components and the identification of these volatiles may allow us to use of floral attractant for biological control of *A. segetum*.

Conventional palynology lacks discriminatory power, is time-consuming and requires advanced skills, thus drastically limiting the number of samples that can be studied [13,19,52]. To overcome these limitations, molecular-based techniques have shown great potential [19,21]. The plant working group of the Consortium for the Barcode of Life recommended the two-locus combination of *rbcL* + *matK* as the plant barcode [53], while other groups have proposed ITS2 as a novel universal barcode for the identification of plant taxa [54,55]. As *matK* is difficult to amplify using universal primer sets and its discrimination power differs between taxonomic groups [55], we concurrently used *rbcL* and ITS2 to identify pollen grains in our study. Yet, pollen DNA barcoding for pollination study applications still remains at the proof-of-concept stage [21]. Furthermore, single pollen DNA is easily contaminated by the ambient environment [3] and incomplete reference databases for DNA barcoding further hamper successful pollen identification [56]. Some scientists continue to advocate the use of classical palynology to study pollen grains on insect bodies [56] and others believe that DNA barcoding will become a powerful method for studying entomophilous pollen transfers at a wide community scale in the future [56]. Our combined use of classical approaches and DNA-based barcoding successfully identified a broad range of pollen types and could thus be recommended for current studies. The study of plant-pollinator ecology is an important field of research, as it sheds light upon the mutually-beneficial relationship between plants and pollinating animals such as Noctuid moths [57]. Our results showed the potential role of *A. segetum* in a range of plant species such as *C. sinensis*, *M. azedarach*, *O. europaea*, *L. lucidum*, *R. pseudoacacia*, *C. echinocarpa* and *C. henryi*. Meanwhile, our molecular-assisted pollen grain analysis provided evidence for the northward spring migration of *A. segetum*. This research provides the basis for a more in-depth assessment of the relationship between *A. segetum* food plant choice and host plant choice by comparing the identity of pollen grains on *A. segetum* moths’ exterior body versus its crop. Furthermore, our work also enables an advanced assessment of the relative importance of pollen and nectar as food resources, particularly in light of the moth’s (long-distance) migratory behavior. Lastly, our study permits the targeting of (area-wide) management interventions in areas and habitats where migratory *A. segetum* populations originate.

## 4. Materials and Methods

### 4.1. Moth Collection

Collection of *A. segetum* long-distance migrants was carried out on Beihuang island (BH, 38°24′ N, 120°55′ E), which is located in the center of the Bohai Strait at a distance of ≈60 km from the mainland to the south and ≈40 km to the north. And the location of this island has been drawn in Guo et al. study [32]. *A. segetum* moths were collected using a vertical-pointing searchlight trap (model JLZ1000BT; Shanghai Yaming Lighting Co. Ltd., Shanghai, China). The searchlight trap was switched on at sunset and switched off at sunrise on all nights from April to October 2014–2017, with the exception of days that exhibited power cuts or when heavy rain occurred. Moths were collected with a nylon net bag (60 mesh) beneath the trap, which was changed manually every 2 h each night. Twenty moths (or all individuals if the total captured was <20) were removed from bags every morning. Captured moths were placed individually into 2 mL tubes and frozen until microscopic examination.

### 4.2. Pollen Examination and SEM Preparation

Pollen was usually found on the proboscis and occasionally on the eye or other parts of the body [9,27]. To determine the presence of pollen, *A. segetum* adult proboscis was dissected and examined at 200× magnification using a stereomicroscope (Olympus SZX16, Pittsburgh, PA, USA). To prevent contamination, a piece of paper towel (9 cm × 9 cm) was placed on the microscope slide and changed with each new sample and all forceps were rinsed with ethyl alcohol after each sample. Pollen grains were isolated from the proboscis, mounted on aluminum stubs, dried in a fume hood and subsequently sputter-coated with gold palladium using a Hummer vacuum coating machine. After coating, each specimen was photographed using a Hitachi S-4800 SEM (Hitachi High-Technologies Co., Tokyo, Japan).

### 4.3. Pollen Lysis and Single Pollen PCR

For DNA extraction from a single pollen grain, we modified the extraction method described by Chen et al. [58]. Under a stereomicroscope, a single pollen grain was picked from the aluminum stubs using a plastic pipette tip (micropipette puller, Sutter Instruments, Novato, CA, USA). Next, the pollen grain was placed in individual PCR tubes that contained 5 μL of lysis solution (0.1 M NaOH, plus 2% Tween-20). Samples were spun briefly before incubation at 95 °C for 17 min 30 s to lyse the pollen grains. After lysis, equi-molars of 5 μL Tris-EDTA (TE) buffer were added to neutralize the samples, which were then spun briefly. The final DNA solution was preserved at −20 °C and was used to amplify plant plastid DNA.

The partial region of chloroplast gene was amplified using universal primers. The nucleotide sequences (5′ to 3′) of the primers as were as follows: primers rbcla forward (ATGTCACCACAAACAGAAAC) and reverse (TCGCATGTACCTGCAGTAGC) [59]; primers rbclb forward (ATGTCACCACAAACAGAAAC) and reverse (GAAACGGTCTCTCCAACGCAT) [60]; primers ITS forward (GACTCTCGGCAACGGATATC) [61] and reverse (TCCTCCGCTTATTGATATGC) [62]. The PCR reaction mixture (25 μL) contained 1 μL extracted DNA, 200 μM of each dNTP, 0.2 μM of each primer, 0.15 μL Ex Taq polymerase (Takara, Dalian, China), 2.5 μL 10× reaction buffer and 19.35 μL nuclease-free water. PCR conditions were an initial denaturation at 94 °C for 5 min; followed by 35 cycles at 94 °C for 30 s, 56 °C or 30 s, 72 °C for 45 s; and a final extension at 72 °C for 10 min. The PCR amplification was performed using a thermal cycler (GeneAmp PCR System 9700, Applied Biosystems, Foster City, CA, USA).

PCR amplification products were separated by electrophoresis on a 1.5% agarose gel containing ethidium bromide and then visualized and photographed under ultraviolet trans-illumination prior to purification. PCR products were purified with a Gel Extraction Kit (Tiangen, Beijing, China) and sub-cloned into pEASY-T3 Cloning Vector (TransGen Biotech, Beijing, China) and the inserts were sequenced with standard M13 primers (Shanghai Sangon, Beijing, China).

### 4.4. Pollen Identification and Characteristics of Pollen Source Plants

SEM photographs are undistorted by the optical interference encountered in light microscopy and thus enable a sound assessment of pollen grain surface sculpture. The pollen grain’s morphological features were identified using modern palynological textbooks and atlases, including of pollen flora of China [63], pollen flora of China woody plants [64] by SEM and pollen flora of China vegetables by SEM [65]; and using palynological literature [9,28,66]; and using online query website, such as university of Arizona SEM’s (available online: http://www.geo.arizona.edu/palynology/sem/semuofa.html) and palynological database (available online: https://www.paldat.org/). Next, species were verified according to their DNA sequences. The species identity of sequences generated in this study was determined using the Basic Local Alignment Search Tool (BLAST, NCBI National Center for Biotechnology Information, available online: https://blast.ncbi.nlm.nih.gov/Blast.cgi) method [49,67]. The BLAST method assesses the identity of a sample based on the best hit of the query sequence, with the E-value for the match being less than a cutoff value. Blast is commonly used as a reliable method of identification and can give accurate identification at the genus [68]. Pollen grains that could be identified and classified to family, genus or species level were used as a reference collection for further identification purposes.

### 4.5. Data Analysis

The rates and the taxa of pollen adhering to *A. segetum* during different migration stages was analyzed using a one-way analysis of variance (ANOVA) in conjunction with Tukey’s honestly significant difference (HSD) test. Differences in the annual mean frequencies of pollen deposits on female and male *A. segetum* moths were analyzed by Student’s *t*-test. Differences in annual frequencies of pollen adherence on female and male *A. segetum* moths and the characteristics of pollen source plants were all compared by using a Chi-square test. All statistical analyses were performed by using SPSS 20.0 (SPSS, Chicago, IL, USA) and all proportion data were logit transformed before being analyzed.

## 5. Conclusions

Palynology or pollen grain identification is useful in studying insect migration and pollination ecology and in shedding light upon different aspects of plant-insect interactions. In this study, we gain a profound understanding of the interactions between the turnip moth *A. segetum* and a broad range of (host plant) species in China. Furthermore, our innovative use of conventional morphology-based pollen identification and DNA metabarcoding permits a rapid, (relatively) straightforward and low-cost identification of a multitude of pollen taxa attached to Noctuid insect body. Our results advance our understanding of the trophic relationship between a (long-distance) migratory Noctuid moth and its host plants over large geographical scales and lays the basis for effective, targeted management of globally-importance agricultural pest.

## Figures and Tables

**Table 1:** Comparative assessment of the degree of taxonomic identification obtained through either molecular or morphology-based approaches, for 40 different types of pollen grains dislocated from *A. segetum* long-distance migrants collected on Behuang Island (Bohai Sea, northeastern China). For each type of pollen grain, the highest level of taxonomic identification is indicated and contrasted between molecular and morphology-based approaches. For each host plant, geographic distribution within China is equally specified.

Pollen Grain Type	Identified Plants	Molecular Identification	Morphology-Based Identification	Geographic Distribution in China
1	*Citrus sinensis*	Sister to *Citrus limon*/*Citrus maxima*/*Citrus sinensis*	*Citrus sinensis*	Zhejiang, Taiwan, Fujian, Jiangxi, Hubei, Hunan, Guangdong, Giangxi, Yunnan, Guihzhou, Sichuan
2	*Heliotropium* L.	Sister to *Heliotropium stenophyllum*/*Heliotropium huascoense*	*Heliotropium* L.	From south to southeast of China
3	Liliaceae	Unidentifiable	Liliaceae	The nationwide distribution
4	Cupressaceae	Unidentifiable	Cupressaceae	The nationwide distribution
5	*Robinia pseudoacacia*	*Robinia pseudoacacia*	*Robinia* L.	Gansu, Qinghai, Neimenggu, Xinjiang, Shanxi, Shaanxi, Hebei, Henan, Shandong et al.
6	Leguminosae	Unidentifiable	Leguminosae	The nationwide distribution
7	*Amorpha fruticosa*	Sister to *Amorpha nana*/*Amorpha fruticosa*	*Amorpha fruticosa*	The nationwide distribution
8	*Cercidium* L.	Sister to *Cercidium andicola*/*Parkinsonia africana*	*Cercidium* L.	The nationwide distribution
9	*Pterocarya rhoifolia*	*Pterocarya rhoifolia*	*Pterocarya* Kunth.	Shandong
10	*Elaeagnus umbellata*	*Elaeagnus umbellata*	*Elaeagnus* L.	North China, east China, southwest of China and Shaanxi, Gansu, Qinghai, Ningxia, Liaoning, Hubei
11	*Fendlera* Engelm. & Gray	Sister to *Fendlera rupicola/Hydrangea quercifolia*	*Fendlera* Engelm. & Gray	The nationwide distribution
12	*Corylus* L.	Sister to *Corylus avellana/Ostryopsis nobilis*	*Corylus* L.	From southwest to northeast of China
13	*Betula* L.	Sister to *Betula pendula*/*Betula alba*	*Betula* L.	The nationwide distribution
14	*Melia azedarach*	*Melia azedarach*	*Melia* L.	South of the Yellow River
15	*Olea europaea*	*Olea europaea*	*Olea* L.	Jiangsu, Anhui, Hubei, Hunan, Guizhou, Sichuan, Yunnan, Guangxi, Guangdong et al.
16	*Ligustrum lucidum*	Sister to *Ligustrum lucidum*/*Forsythia suspensa*	*Ligustrum* L.	Jiangsu, Zhejiang, Jiangxi, Anhui, Shandong, Hubei, Hunan, Guizhou, Sichuan, Fujian, Guangxi, Guangdong
17	Brassicaceae	Unidentifiable	Brassicaceae	The nationwide distribution
18	*Brassica* L.	Sister to *Brassica rapa*/*Brassica napus*/*Brassica oleracea*/*Brassica juncea*	*Brassica* L.	The nationwide distribution
19	Alliaceae	Unidentifiable	Alliaceae	The nationwide distribution
20	*Pinus* L.	Sister to *Pinus tabuliformis*/*Pinus thunbergii*/*Pinus densata*/*Pinus hwangshanensis*/*Pinus kesiy*a/*Pinus yunnanensisu*	*Pinus* L.	The nationwide distribution
21	Pinaceae	Unidentifiable	Pinaceae	The nationwide distribution
22	*Helianthus* L.	Sister to *Helianthus annuus*/*Helianthus argophyllus*/*Helianthus debilis/Helianthus tuberosus*/*Helianthus pauciflorus*/*Helianthus mollis*/*Helianthus petiolaris*/*Helianthus maximiliani*	*Helianthus* L.	The nationwide distribution
23	*Artemisia* L.	Sister to *Artemisia gmelinii*/*Artemisia vulqaris*	*Artemisia* L.	The nationwide distribution
24	Asteraceae	Unidentifiable	Asteraceae	The nationwide distribution
25	*Gnaphalium* L.	Sister to *Gnaphalium uliqinosum*/*Gnaphalium affine*	*Gnaphalium* L.	The nationwide distribution
26	*Chrysanthemum* L.	Sister to *Chrysanthemum mutellinum*/*Chrysanthemum indicum*/*Chrysanthemum x morifoliu*m/*Chrysanthemum lavandulifolium*/*Chrysanthemum maximum*	*Chrysanthemum* L.	The nationwide distribution
27	*Dendromecon* Benth.	Unidentifiable	*Dendromecon* Benth.	The nationwide distribution
28	*Eschscholtzia* Cham.	Unidentifiable	*Eschscholtzia* Cham.	The nationwide distribution
29	*Polygala* L.	Sister to *Polygala setacea*/*Polygala alba*/*Polygala qalapageia*/*Polygala sancti-qeorqii*	*Polygala* L.	The nationwide distribution
30	*Castanea henryi*	Sister to *Castanea sativa*/*Castanea mollissima*/*Castanea henryi*	*Castanea henryi*	Jiangsu, Zhejiang, Jiangxi, Anhui, Shandong, Hubei, Hunan, Guizhou, Sichuan, Fujian, Guangxi, Guangdong
31	*Castanopsis echinocarpa*	Sister to *Castanopsis echinocarpa*/*Castanopsis carlesii*	*Castanopsis echinocarpa*	Southern of Yunnan province, southeast of the Tibet autonomous region
32	*Ailanthus* Desf.	Sister to *Ailanthus fordii*/ *Ailanthus altissima*/*Ailanthus excelsa*	*Ailanthus* Desf.	The nationwide distribution
33	Rosaceae	Unidentifiable	Rosaceae	The nationwide distribution
34	Rosaceae	Unidentifiable	Rosaceae	The nationwide distribution
35	*Adenophora trachelioides*	Sister to *Adenophora erecta*/*Adenophora remotiflora/*	*Adenophora trachelioides*	Liaoning, Hebei, Shandong, Jiangsu, Anhui, Zhejiang
		*Adenophora trachelioides*		
36	*Chenopodium album*	Sister to *Chenopodium serotinum*/*Chenopodium album*/*Chenopodium acuminatum*/*Chenopodium quinoa*	*Chenopodium album*	The nationwide distribution
37	*Gaura* L.	Sister to *Gaura coccinea*/*Oenothera suffrutescens*	*Gaura* L.	North of China
38	*Galium* L.	Sister to *Galium boreale*/*Galium sp.*	*Galium* L.	The nationwide distribution
39	*Pilea Lindl.*, *nom. conserv.*	Sister to *Pilea depressa*/*Pilea plataniflora*/*Pilea microphylla*	*Pilea Lindl.*, *nom. conserv.*	The nationwide distribution
40	Lauraceae	Unidentifiable	Lauraceae	The nationwide distribution

**Table 2:** Annual disaggregated data reflecting the percentage of *A. segetum* moths with adhering pollen grains and the ensuing level of taxonomic resolution of their identification.

No./%	2014	2015	2016	2017	Total
No. adults examined	773	492	628	673	2566
No. with pollen	72	132	158	75	437
% with pollen	9.31	26.83	25.16	11.14	17.03
No. taxa	15	27	24	18	40
No. families	14	17	19	13	26
No. genera	5	13	9	6	18
No. species	7	8	8	7	12

**Table 3:** Annual patterns in the level of pollen grain adherence among female and male adults of *A. segetum*, sampled over a 2014–2017 time period in Beihuan Island (Bohai Sea, northeastern China).

Year	Female	Male	The Value of Test	
	No. (%) of Moths Contaminated			
2014	50 (9.52)	22 (8.87)	χ^2^	0.085
			*df*	1
			*p*	0.771
2015	68 (31.19)	64 (23.36)	χ^2^	3.796
			*df*	1
			*p*	0.051
2016	68 (23.69)	90 (26.39)	χ^2^	0.603
			*df*	1
			*p*	0.437
2017	38 (10.86)	37 (11.46)	χ^2^	0.061
			*df*	1
			*p*	0.805
2014–2017	350 (25.36)	323 (27.23)	*t*	0.165
			*df*	6
			*p*	0.875

**Table 4:** Relative seasonal occurrence of different types of pollen grains, as adhering to migrant *A. segetum* adults sampled in Beihuang island (Bohai sea) over 2014–2017.

Family	Early-Season (May–June)	Mid-Season (July–August)	Late-Season (September–October)	Overall Total
Pinaceae	31.9	7.23	4.1	19.45
Leguminosae	10.34	1.2		5.72
Oleaceae	9.48			5.03
Brassicaceae	8.62	31.33		10.53
Meliaceae	8.19			4.35
Rosaceae	7.76	4.82		5.03
Elaeagnaceae	4.74			2.52
Fagaceae	4.74	7.23		3.89
Boraginaceae	3.02			1.6
Simaroubaceae	2.59			1.37
Alliaceae	2.16			1.14
Cupressaceae	1.72			0.92
Rutaceae	1.29			0.69
Asteraceae	0.86	7.22	68.85	21.28
Papaveraceae	0.86			0.46
Polygalaceae	0.43			0.23
Juglandaceae	0.43			0.23
Betulaceae	0.43	1.2		0.46
Saxifragaceae	0.43			0.23
Onagraceae		16.87		3.2
Chenopodiaceae		16.87	9.02	5.72
Campanulaceae		3.61		0.69
Rubiaceae		2.41		0.46
Urticaceae			14.75	4.12
Lauraceae			3.28	0.92

## Supplementary Materials

Supplementary materials can be found at http://www.mdpi.com/1422-0067/19/2/567/s1. Text S1: The DNA sequences of the examined pollen species.
