# Supplementary material for: Molecular-Assisted Pollen Grain Analysis Reveals Spatiotemporal Origin of Long-Distance Migrants of a Noctuid Moth
Source: Int J Mol Sci. 2018 Feb 13;19(2):567. doi: 10.3390/ijms19020567 (PMC5855789; doi:10.3390/ijms19020567)
Supplement: Supplementary file 1 [file ijms-19-00567-s001.pdf]

Supplementary Materials: Molecular-Assisted Pollen Grain Analysis Reveals Spatiotemporal Origin of Long-Distance Migrants of a Noctuid Moth

Hong Chang, Jianglong Guo, Xiaowei Fu, Yongqiang Liu, Kris AG Wyckhuys, Youming Hou and Kongming Wu

Text S1. The DNA sequences of the examined pollen species.
>1
TAAAGCGAGTGTTGGATTCAAGGCCGGTGTTAAAGATTATAAATTGACTTATTATACTCCTGACTATGTA
ACCAAAGATACTGATATCTTGGCAGCATTCCGAGTAACTCCTCAGCCCGGAGTTCCACCCGAGGAAGC
GGGGGCTGCGGTAGCTGCGGAATCTTCTACTGGTACCTGGACAGCTGTGTGGACCGATGGGCTTACCA
GCCTTGATCGTTACAAAGGGCGATGCTACAACATTGAGCCCGTTGCTGGAGAAGAGAATCAATATATAT
GTTATGTAGCTTACCCGTTAGACCTTTTTGAAGAAGGTTCTGTTACTAACATGTTTACTTCCATTGTGGGT
AATGTATTTGGTTTCAAAGCACTGCGCGCTCTACGTCTAGAGGATCTACGAATCCCTCCTGCGTATACTA
AAACTTTCCAAGGCCCGCCTCACGGCATCCAAGTTGAGAGAGATAAATTGAACAAGTATGGCCGTCCC
CTGTTGGGATGTACTATTAAACCTAAACTGGGGTTATCCGCGAAGAATTATGGTAGGGCGGTTTATGAAT
GTCTACGTGGTGGACTTGACTTTACCAAAGATGATGAGAACGTGAACTCCCAACCATTTATGCGTTGGA
GGGACCGTTTCTTATTTTGTGCGGAAGCTCTTTATAAAGCGCAAGCTGAAACAGGTGAAATCAAAGGTC
ATTACTTGAAT
>2
TCCTCCGCTTATTGATATGCTTAAACTCAGCGGGTAATCCCGCCTGACCTGGGGTCGCGGTCGTAGCGCG
CGGCTCCGCTCGGGAGACTTGCACGTCAGGGTCTCGTGGGTCTCCGACGCACGGCGGGGAATCGCACG
GCAGCACGCGAGTTGAAATGTCCAACCACCACTTGCCGTGACGTCCATCGTGCCGGACTCGCATTTCG
GCCAGCCGCGCACGGGGCACGGGAGGCCACAATCCGCGCCCCTCCGGAGGAGGGGGCGACGCGATG
CGTGACGCCCAGGCAGACGTGCCCTCGGCCTGACGGCTTCGGGCGCAACTTGCGTTCAAAGACTTGAT
GGTTCACGGGATTCTGCAATTCACACCAAGTATCGCATTTCGCTACGTTCTTCATCGATGCGAGAGCCG
AGATATCCGTTGCCGAGAGTC
>5
TAAAGCAAGTGTTGGGTTCAAAGCTGGTGTTAAAGATTATAAATTGACTTATTATACTCCTGACTATGAA
ACCAAAGATACTGATATCTTGGCAGCATTCCGAGTAACTCCTCAACCTGGAGTTCCGCCTGAAGAAGC
AGGTGCAGCGGTAGCTGCCGAATCTTCCACTGGTACATGGACAACTGTGTGGACCGATGGGCTTACCA
GTCTTGATCGTTACAAAGGACGATGCTACCACATCGAGCCCGTTGCTGGAGAAGAAAGTCAATTTATTG
CCTATGTAGCTTATCCCTTAGACCTTTTTGAAGAAGGTTCTGTTACTAACATGTTTACTTCCATTGTAGGT
AATGTATTTGGGTTCAAGGCCCTGCGCGCTCTGCGTCTGGAGGATTTGCGAATCCCTACTGCTTACATTA
AAACTTTTCAGGGCCCACCTCACGGAATCCAAGTTGAGAGAGATAAATTGAACAAGTATGGCCGCCCC
CTATTGGGATGTACTATTAAACCTAAATTGGGGTTATCCGCTAAGAATTATGGTAGAGCAGTTTATGAAT
GTCTCCGCGGGGGACTTGATTTTACTAAAGATGATGAAAATGTGAATTCCCAACCATTT
>7
TCCTCCGCTTATTGATATGCTTAAACTCAGCGGGTAGCCCCGCCTGACCTGAGGTCCCATTGTGGGCGAT
CGAATCACCCCCGTGGGTCACGAAGTCCACTCCAGGTGGATGCACGCGCGACTGGCCTCGAGCTCTTT
ACTCAACCACCATCTGTCACGGTGCACTCAGCCACGGACTCGATTTTCAACCAACCGTGAGGCTGTGC
TCACGGGAGGCCAGCATTCACCCCCAGCTAGATGCCTGGCATGAGGCATTGGCGTTGGGGCAACGATG
TGTGACACCCAGGCAGGCGTGCCCTCAGCCTAATGGCTTCGGGCGCAACTTGCGTTCAAAGACTCGAT
GGTTCACGGGATTCTGCAATTCACACCAAGTATCGCATTTCGCTACGTTCTTCATCGATGCAAGAGCCG
AGATATCCGTTGCCGAGAGTC
>8
TAAAGCAAGTGTTGGNTTCAAAGCTGGTGTTAAAGATTATAAATTGACTTATTATACTCCTGACTATGAA
ACCAAAGATACTGATATCTTGGCAGCATTCCGAGTAACTCCTCAACCTGGAGTTCCGCCTGAAGAAGC
AGGTGCCGCGGTAGCTGCTGAATCTTCTACTGGTACATGGACAACTGTGTGGACCGATGGGCTTACCAG
TCTTGATCGTTACAAAGGGCGATGCTACCACATCGAGCCCGTTGCTGGAGAAGAAAATCAATATATTGC
TTATGTAGCTTATCCCTTAGACCTTTTTGAAGAAGGTTCTGTTACTAACATGTTTACTTCCATTGTGGGTA

ATGTATTTGGGTTCAAGGCCCTGCGCGCCCTACGTCTGGAGGATTTGCGAATCCCTACTTCTTATACTAA
AACTTTCCAAGGTCCGCCTCACGGCATCCAAGTTGAGAGAGATAAATTGAACAAGTACGGGCGTCCCC
TATTGGGATGTACTATTAAACCTAAATTGGGGTTATCCGCTAAGAATTACGGTAGAGCAGTTTATGAATG
TCTCCGCGGTGGACTTGATTTTACCAAAGATGATGAGAATGTGAATTCCCAACCATTTATGCGTTGGAG
AGACCGTTTCTTATTTTGTGCCGAAGCAATTTATAAAGCACAGGCCGAAACAGGTGAAATCAAAGGGC
ATTACTTGAAT
>9
TAAAGCAAGTGTTGGATTCAAAGCTGGTGTTAAAGATTATAAATTGACTTATTATACTCCTGAATATCAA
ACCAAAGATACTGATATCTTGGCAGCGTTCCGAGTAAGCCCTCAACCTGGAGTTCCGCCTGAGGAAGC
AGGGGCAGCAGTAGCTGCTGAATCTTCTACTGGTACATGGACAACTGTGTGGACCGATGGGCTTACTAG
TCTTGATCGTTACAAAGGACGATGCTACCACATCGAGCCAGTTGCTGGAGAAGAAAATCAATTTATTGC
TTATGTAGCTTACCCCTTAGACCTTTTTGAAGAAGGTTCTGTTACTAACATGTTTACTTCCATTGTGGGTA
ATGTATTTGGATTCAAGGCCCTGCGCGCTCTACGTCTGGAGGATTTGCGAATCCCTCCTGCTTATTCTAA
AACTTTCCAAGGCCCGCCTCATGGAATCCAAGTTGAGAGAGATAAATTAAACAAGTATGGCCGCCCTCT
ATTGGGATGTACTATTAAACCTAAATTGGGATTATCCGCTAAGAATTACGGTAGAGCAGTTTATGAATGT
CTCCGCGGTGGGCTTGATTTTACCAAAGATGATGAGAACGTAAATTCCCAACCATTT
>10
TCCTCCGCTTATTGATATGCTTAAACTCAGCGGGTAGCCCCGCCTGACCTGGGGTCGCATCGAAAGCAT
CCATGTTGGATGCGTTGGGGTCCTTGCATCGTGCACGACCAAAACCGACGACGGGGCACCGAAGGTTC
GACAACCACAATTGTCGTGGCATTGGTCGCCAATGACTAGAATTTGGGCCAACCACAAGGCAAAGCTC
ATGGGAAGCCAATGTACGCCTTCGCAACCGACGCAGCCTCGAAGGCACGTGGTGTTAGGAGGGCAAC
GGTGTGTGACGCCCAGGCAGACGTGCCCTCAACCGGATGGCTTCGGGCGCAACTTGCGTTCAAAGACT
CGATGGTTCACGGGATTCTGCAATTCACACCAAGTATCGCATTTCGCTACGTTCTTCATCGATGCGAGAG
CCGAGATATCCGTTGCCGAGAGTC
>11
TAAAGCAAGTGTTGGATTCAAAGCTGGTGTTAAAGATTACAAATTGACTTATTATACTCCTGACTATGAA
ACCAAAGATACTGATATCTTGGCAGCATTCCGAGTAACTCCTCAACCTGGAGTTCCACCTGAAGAAGC
AGGGGCCGCGGTAGCTGCCGAATCTTCTACTGGTACATGGACAACTGTGTGGACCGATGGACTTACCA
GCCTTGATCGTTACAAGGGGCGATGCTACCACATCGAGCCTGTTGCTGGAGAAGAAAATCAATTTATTG
CTTATGTAGCTTACCCATTAGACCTTTTTGAAGAAGGTTCTGTTACTAACATGTTTACTTCCATTGTGGGT
AATGTATTTGGGTTCAAAGCCCTGCGTGCTCTACGTCTGGAAGATCTGCGAATCCCTGTTGCTTATGTTA
AAACTTTCCAAGGCCCGCCTCATGGCATCCAAGTTGAGAGAGATAAATTGAACAAGTATGGTCGTCCC
CTGTTGGGATGTACTATTAAACCGAAATTGGGGTTATCTGCTAAAAACTACGGTAGAGCAGTTTATGAAT
GTCTCCGCGGTGGACTTGATTTTACCAAAGATGATGAGAACGTGAACTCCCAACCATTTATGCGTTGGA
GAGACCGTTTCTTATTTTGTGCAGAAGCAATTTATAAAGCACAGGCTGAAACGGGTGAAATCAAAGGG
CATTACTTGAAT
>12
GACTCTCGGCAACGGATATCTCGGCTCTCGCATCGATGAAGAACGTAGCGAAATGCGATACTTGGTGTG
AATTGCAGAATCCCGCGAATCATCGAGTCTTTGAACGCAAGTTGCGCCCGAAGCCATTTGGTCGAGGG
CACGTCTGCCTGGGTGTCACGCATCGTTGCCCCCAACCCCATTGCTTCTCCAAGAGACGAGGGCGGTTT
GTGGGGCGGACATTGGCCTCCCGTGAGCTTTTGATTGCGGCTGGCCTAAAAGGGAGTCCTCGGCGACG
AGCGCCACGACAATCGGTGGTTGACAAACCCTCGTGTCCCGTCGTGCGTGCCTTGTCGCTCATCTCGTG
CTCTGTGACCCTATAGCGTCGCGCTTGCGACTCTTCCAACGCGACCCCGGGTCAGGCGGGACTACCCGC
TGAATTTAAGCATATCAATAAGCGGAGGA
>13
GACTCTCGGCAACGGATATCTCGGCTCTCGCATCGATGGAGAACGTAGCGAAATGCGATACTTGGTGTG
AATTGCAGAATCCCGCGAATCATCGAGTCTTTGAACGCAAGTTGCGCCCGAAGCCACCTGGCCGAGGG
CACGTCTGCCTGGGTGTCACGCATCGTTGCCCCCAACCCCATCTCCTTGCAAAGGAACGAGGGGGCCT
GTGGGGCAGAAATTGGCCTCCCGTGAGCTCATGCATGCGGTTGGCCTAAAAGCGAGTCCTCGGCGACG
CGCGCCACGACAATCGGTGGTTGTCAAACCCTCGTGTCCCGTCGTGCGTGCCGCGTCGCTCATCGTGTG
CTCCTTGACCCTGCTGTGTCGCGCTAGCGACGCTTCCAACGCGACCCCAGGTCAGGCGGGACTACCCG
CTGAATTTAAGCATATCAATAAGCGGAGGA
>14
GACTCTCGGCAACGGATATCTCGGCTCTCGCATCGATGAAGAACGTAGCGAAATGCGATACTTGGTGTG

AATTGCAGAATCCCGTGAACCATCGAGTCTTCGAACGCAAGTTGCGCCCCAAGCCGTTAGGCCGAGGG
CACGTCTGCCTGGGTGTCACGCATCGTTGCCCCCCCGCACAATCCCCTCTCGGGGGAAGAGGTGTCGG
GCGGGCGGAGACTGGCCTCCCGTGCGCTCCGCGCTCGCGGTTGGCCGAAATTCGAGTCCTTCGGCGAC
CGGGCCGCGACGATCGGTGGTGAGAACAAGCCTCTCGAGCTCCAGTCGCGCGCCCGCGCCTCCGTGC
GAGGGACTCGCGGACCCTTGTGCACGCCCCTCCGGGCGATGCTCGCTTCGCGACCCCAGGTCAGGCGG
GACTACCCGCTGAGTTTAAGCATATCAATAAGCGGAGGA
>15
TAAAGCAAGTGTTGGATTCAAAGCGGGTGTTAAAGAGTACAAATTGACTTATTATACTCCTGAATACGA
AACCAAAGATACTGATATCTTGGCAGCATTCCGAGTAACTCCTCAACCTGGAGTTCCGCCTGAAGAAG
CAGGGGCTGCGGTAGCTGCCGAATCTTCTACTGGTACATGGACAACTGTGTGGACCGATGGACTTACCA
GCCTTGATCGTTACAAAGGGCGATGCTACCATATTGAGCCCGTTCCTGGAGAAGCAGATCAATATATCT
GTTATGTAGCTTACCCTTTAGACCTTTTTGAAGAAGGTTCTGTTACTAACATGTTTACTTCCATTGTAGGT
AATGTATTTGGGTTCAAAGCCCTGCGTGCTCTACGTCTGGAAGATCTGCGAATCCCTACTGCTTATATTA
AAACTTTCCAAGGCCCGCCTCATGGGATCCAAGTTGAGAGAGATAAATTGAACAAGTATGGTCGTCCC
CTGTTGGGATGTACTATTAAACCAAAATTGGGGTTATCTGCTAAAAACTATGGTAGAGCAGTTTATGAAT
GTCTTCGCGGTGGACTTGATTTTACCAAAGATGATGAGAACGTGAACTCCCAGCCATTTATGCGTTGGA
GAGATCGTTTCTTATTTTGTGCCGAAGCACTTTATAAAGCACAGGCTGAAACAGGTGAAATCAAAGGG
CATTACTTGAAT
>16
TAAAGCAAGTGTTGGATTCAAAGCGGGTGTTAAAGAGTACAAATTGACTTATTATACTCCTGAATACGA
AACCAAAGATACTGATATCTTGGCAGCATTCCGAGTAACTCCTCAACCTGGAGTTCCGCCTGAAGAAG
CAGGGGCTGCGGTAGCTGCCGAATCTTCTACTGGTACATGGACAACTGTGTGGACCGATGGACTTACCA
GCCTTGATCGTTACAAAGGGCGATGCTACCATATTGAGCCCGTTCCTGGAGAAGCAGATCAATATATCT
GTTATGTAGCTTACCCTTTAGACCTTTTTGAAGAAGGTTCTGTTACTAACATGTTTACTTCCATTGTAGGT
AATGTATTTGGGTTCAAAGCCCTGCGTGCTCTACGTCTGGAAGATCTGCGAATCCCTCCTGCTTATATTA
AAACTTTCCAAGGCCCGCCTCATGGGATCCAAGTTGAGAGAGATAAATTGAACAAGTATGGTCGTCCC
CTGTTGGGATGTACTATTAAACCAAAATTGGGGTTATCTGCTAAAAACTATGGTAGAGCAGTTTATGAAT
GTCTTCGCGGTGGACTTGATTTTACCAAAGATGATGAGAACGTGAACTCCCAGCCATTT
>18
AAATGGTTGGGAGTTCACATTCTCATCATCTTTGGTAAAATCAAGTCCACCACGTAGACATTCATAAACT
GCTCTACCACAGTTCTTCGCGGATAACCCCAATTTAGGTTTAATAGTACATCCTAATAGGGGACGTCCAT
ACTTGTTCAATTTCTCTCTTTCAACTTGGATACCATGAGGTGGTCCCTGGAAAGTTTGAGTATAAGCAGG
AGGGATTCGCAGATCCTCTAGACGTAGAGCAGCCAGGGCTTTGAACCCAAATACATTACCCACAATCG
AAGTAAACATGTTAGTAACCGAACCTTCTTCAAAAAGGTCTAAAGGGTAAGCTACATAAGCAATAAAT
TGATTTTCTTCTCCTGGAACGGGCTCGATGTGGTAGCATCGTCCTTTGTAACGATCAAGGCTGGTAAGCC
CATCGGTCCACACAGTTGTCCATGTACCAATAGAAGATTCAGCAGCTACCGCAGCCCCTGCTTCTTCAG
GTGGAACTCCGGGTTGAGGAGTTACTCGGAATGCTGCCAAGATATCAGTATCCTTGGTTTCATATTCAGG
AGTATAATAAGTCAATTTATACTCTTTTTAACACCAGCTTTGAATCCAACACTTGCTTT
>20
AAAGCTAGTGTCGGATTCAAAGCTGGTGTTAAAGATTACAGATTAACTTATTATACTCCTGAATATCAGA
CCAAAGATACAGATATCTTGGCAGCATTCCGAGTAACTCCTCAACCCGGGGTGCCACCTGAAGAAGCG
GGAGCAGCAGTAGCTGCTGAATCTTCCACCGGTACATGGACCACTGTTTGGACCGATGGACTTACTAGT
CTCGATCGTTACAAGGGGCGATGCTATGACATCGAGCCCGTTCCTGGAGAGGAGACTCAATTTATTGCC
TATGTAGCTTACCCCTTAGACCTTTTCGAAGAAGGTTCTGTTACTAACTTGTTCACTTCCATTGTAGGTAA
TGTATTTGGATTCAAGGCCTTACGGGCTCTACGTTTGGAAGATTTGCGGATTCCCCCTTCTTATTCCAAA
ACTTTTCAGGGTCCACCTCATGGTATCCAAGTTGAAAGAGATAAATTGAACAAATATGGTCGTCCTTTAT
TGGGATGTACTATCAAACCAAAATTGGGTCTATCAGCCAAAAACTATGGTAGAGCAGTTTACGAATGTC
TCCGTGGTGGACTCGATTTTACTAAGGATGATGAGAACGTAAATTCCCAACCATTCATGCGCTGGAGAG
ATCGTTTTGTCTTTTGTGCGGAAGCCCTTAATAAGGCTCAGGCTGAGACGGGTGAAATTAAGGGACATT
ATTTGAAT
>22
TAAAGCAAGTGTTGGATTCAAAGCTGGTGTTAAAGATTATAAATTGACTTATTATACTCCTGAATATGAA
ACCAAGGATACTGATATCTTGGCAGCATTTCGAGTAACTCCTCAACCTGGAGTTCCGCCTGAAGAAGCA
GGGGCCGCAGTAGCTGCCGAATCTTCTACTGGTACATGGACAACTGTATGGACCGATGGACTTACCAGC

CTTGACCGTTACAAAGGCCGATGCTATGGACTTGAGCCTGTTCCTGGAGAAGACAATCAATTTATTGCTT
ATGTAGCGTACCCATTAGACCTTTTTGAAGAAGGTTCTGTTACTAACATGTTTACTTCCATTGTAGGTAAT
GTATTTGGGTTCAAAGCCCTGCGTGCTCTACGTCTGGAAGATTTGCGAATCCCGACTGCGTATGTTAAAA
CTTTCGACGGTCCGCCTCACGGTATCCAAGTTGAAAGAGATAAATTGAACAAGTATGGTCGTCCCCTGT
TGGGATGTACTATTAAACCGAAATTGGGGTTATCCGCTAAAAACTACGGTAGAGCTTGTTATGAATGTCT
TCGTGGTGGCCTTGATTTTACTAAAGATGATGAGAACGTGAACTCCCAACCATTT
>23
TAAAGCAAGTGTTGGATTCAAAGCTGGGGTTAAAGATTATAAATTGACTTATTATACTCCTGAGTATGAA
ACCAAGGATACTGATATCTTGGCAGCATTTCGAGTAACTCCTCAACCTGGAGTTCCGCCTGAAGAAGCA
GGGGCCGCAGTAGCTGCCGAATCTTCTACTGGTACATGGACAACTGTGTGGACCGATGGACTTACGAG
CCTTGATCGTTACAAAGGGCGATGCTATGGAATTGAGCCTGTTCCTGGAGAAGAGAATCAATATATTTG
CTATGTAGCTTACCCATTAGACCTTTTTGAAGAAGGTTCTGTTACTAACATGTTTACTTCCATTGTAGGTA
ACGTATTTGGTTTCAAAGCCCTGCGTGCTCTACGTCTGGAAGATTTGCGAATTCCTACTGCGTATGTTAA
AACTTTCCAAGGTCCGCCTCACGGTATCCAAGTTGAAAGAGATAAATTGAACAAGTATGGTCGTCCTCT
GTTGGGATGTACTATTAAACCTAAATTGGGGTTATCCGCTAAAAACTACGGTAGAGCTGTTTATGAATGT
CTTCGTGGTGGCCTTGATTTTACTAAAGATGATGAGAACGTAAACTCCCAACCATTTATGCGTTGGAGA
GACCGTTTCTTATTTTGTGCCGAAGCTATTTTTAAATCACAAGCTGAAACAGGTGAAATCAAAGGGCAT
TACTTGAAT
>25
TAAAGCAAGTGTTGGATTCAAAGCAGGTGTTAAAGATTATAAATTGACTTATTATACTCCTGAGTATGAA
ACCAAGGATACTGATATCTTGGCAGCATTTCGAGTAACTCCTCAACCTGGAGTTCCGCCTGAAGAAGCA
GGGGCCGCAGTAGCTGCCGAATCTTCTACTGGTACATGGACAACTGTGTGGACCGATGGACTTACGAG
CCTTGATCGTTACAAAGGACGATGCTATGGAATCGAGCCTGTTCCTGGAGAAGAAAATCAATATATTGC
TTATGTAGCTTACCCATTAGACCTTTTTGAAGAAGGTTCTGTTACTAACATGTTTACTTCCATTGTAGGTA
ATGTATTTGGGTTCAAAGCCCTGCGTGCTCTACGTTTGGAAGATTTGCGAATCCCTACTGCGTATGTTAA
AACTTTCCAAGGTCCGCCTCACGGCATCCAAGTTGAGAGAGATAAATTGAACAAGTATGGTCGTCCTCT
GTTGGGATGTACTATTAAACCGAAATTGGGGTTATCCGCTAAAAACTACGGTAGAGCTGTTTATGAATGT
CTTCGTGGTGGCCTTGATTTTACTAAAGATGATGAGAACGTGAACTCCCAACCATTTATGCGTTGGAGA
GACCGTTTCTTATTTTGTGCCGAAGCTATTTATAAATCACAAGCTGAAACAGGGGAAATCAAAGGGCAT
TACTTAAAT
>26
GGAAGGTTTGCATCAAAGCTGGTGTTAAGATTATAAATTGACTTATTATACTCCTGAGTATCGAAACCAA
AGGATACTGATATCTTGGCAGCATTTCGAGTAACTCCTCAACCTGGAGTTCCGCCTGAAGAAGCAGGG
GCCGCAGTAGCTGCCGAATCTTCTACTGGTACATGGACAACTGTGTGGACCGATGGACTTACGAGCCTT
GATCGTTACAAAGGACGATGCTATCACAATTGAGCCTGTTCTTGGAGAAGAGAATCAATATATTTGCTAT
GTAGCTTACCCATTAGACCTTTTTGAAGAAGGTTCTGTTACTAACATGTTTACTTCCATTGTAGGTAACGT
ATTTGGTTTCAAAGCCCTGCGTGCTCTACGTCTGGAAGATTTGCGAATTCCTACTGCTTATGTTAAAACT
TTCCAAGGTCCGCCTCACGGTATCCAAGTTGAAAGAGATAAATTGAACAAGTATGGTCGTCCTCTGTTG
GGATGTACTATTAAACCTAAATTGGGATTATCCGCTAAAAACTACGGTAGAGCTGTTTATGAATGTCTTC
GTGGTGGCCTTGATTTTACTAAAGATGATGAGAACGTGAACTCCCAACCATTTATGCGTTGGAGAGAAC
CGTTTCATG
>29
TAAAGCAAGTGTTGGATTCAAAGCTGGTGTTAAAGATTATAAATTGACTTATTATACTCCTGACTATGAA
ACCAAAGATACTGATATTTTGGCAGCATTCCGAGTAACCCCCCAACCAGGAGTTCCGCCTGAAGAAGC
GGGGGCCGCGGTAGCTGCTGAATCTTCTACTGGTACATGGACAACTGTGTGGACTGACGGGCTTACGA
GTCTTGATCGTTACAAAGGACGATGCTACCACATCGAGTCTGTTGCTGGAGAAGAAAATCAATATATTG
CTTATGTAGCTTATCCCTTAGACCTTTTTGAAGAAGGTTCTGTTACTAACATGTTTACTTCCATTGTGGGA
AATGTATTTGGGTTCAAGGCTCTGCGCGCTCTACGTTTGGAGGATTTGCGAATCCCACCTGCATATACTA
AAACTTTCCAAGGTCCGCCCCACGGCATTCAAGTTGAGAGAGACAAATTAAACAAGTATGGTCGCCCC
CTTTTGGGATGTACAATTAAACCTAAATTAGGGTTATCTGCTAAGAATTACGGTAGAGCTGTTTATGAAT
GTCTCCGCGGTGGACTTGATTTTACCAAAGATGATGAGAACGTGAATTCCCAACCATTTATGCGTTGGA
GAGACCGTTTCTTATTTTGTGCAGAAGCAATTTATAAAGCACAGGCGGAAACGGGTGAAATCAAAGGG
CATTACTTGAAT
>30

TAAAGCAAGTGTTGGATTCAAAGCTGGTGTTAAAGATTATAAATTGACTTATTATACTCCTGACTATCAA
ACCAAAGATACTGATATCTTGGCAGCCTTCCGAGTAACTCCTCAACCTGGAGTTCCGCCGGAGGAAGC
AGGGGCCGCGGTAGCTGCTGAATCTTCCACTGGGACATGGACAACTGTGTGGACTGACGGGCTTACCA
GTCTTGATCGTTACAAAGGACGATGCTACCACATCGAGCCGGTTGCTGGAGAAGAAAATCAATTTATTG
CTTATGTAGCTTACCCCTTAGACCTCTTTGAAGAAGGTTCTGTTACTAACATGTTTACTTCCATTGTGGGT
AATGTATTTGGATTCAAGGCCCTGCGCGCTCTACGTCTGGAGGATTTGCGAATCCCTACTTCTTATTCTAA
AACTTTCCAAGGTCCGCCTCATGGCATCCAAGTTGAGAGGGATAAATTAAACAAGTATGGCCGCCCCCT
ATTAGGATGTACTATTAAACCTAAATTGGGATTATCCGCTAAGAATTACGGTAGAGCAGTTTATGAATGT
CTCCGCGGTGGGCTTGATTTTACCAAAGATGATGAGAACGTTAATTCCCAACCATTTATGCGTTGGAGA
GACCGTTTCCTATTTGTGCCGAAGCAATTTATAAAGCGCAGGCTGAAACAGGTGAAATCAAGGGGCA
TTACTTGAA
>31
TAAAGCAAGTGTTGGATTCAAAGCTGGTGTTAAAGATTATAAATTGACTTATTATACTCCTGACTATCAA
ACCAAAGATACTGATATCTTGGCAGCCTTCCGAGTAACTCCTCAACCTGGAGTTCCGCCCGAGGAAGC
AGGGGCCGCGGTAGCTGCTGAATCTTCCACTGGGACATGGACAACTGTGTGGACTGACGGGCTTACCA
GTCTTGATCGTTACAAAGGACGATGCTACCACATCGAGCCGGTTGCTGGAGAAGAAAGCCAATTTATTG
CTTATGTAGCTTACCCCTTAGACCTCTTTGAAGAAGGTTCTGTTACTAACATGTTTACTTCCATTGTGGGT
AATGTATTTGGATTCAAGGCCCTGCGCGCTCTACGTCTGGAGGATTTGCGAATCCCTACTTCTTATTCTAA
AACTTTCCAAGGTCCGCCTCATGGCATCCAAGTTGAGAGGGATAAATTAAACAAGTATGGCCGCCCCCT
ATTAGGATGTACTATTAAACCTAAATTGGGATTATCCGCTAAGAATTACGGTAGAGCAGTTTATGAATGT
CTCCGCGGTGGGCTTGATTTTACCAAAGATGATGAGAACGTTAATTCCCAACCATTT
>32
ACAGAAACTAAAGCAAGTGTTGGATTCAAAGCCGGTGTTAAAGATTATAAATTGACTTATTATACTCCT
GAATATGTAACCAAAGATACTGATATCTTGGCAGCATTCCGAGTAACTCCCCAACCCGGAGTTCCGCCC
GAGGAAGCAGGGGCAGCGGTAGCTGCGGAATCTTCTACTGGTACATGGACAACTGTGTGGACCGATGG
GCTTACCAGCCTTGATCGTTACAAAGGACGATGCTACAACATTGAGCCCGTTGCTGGAGAAGAAAATC
AATATATATGTTATGTAGCTTAYCCCTTAGACCTTTTTGAAGAAGGTTCTGTTACTAACATGTTTACTTCGA
TTGTGGGTAATGTATTTGGGTTCAAAGCCCTGCGCGCTCTACGTCTAGGAGGATCTACGAATCCCTCCTG
CGTATTCGAAAACTTTCCAAGGYCCGCCTCACGGTATCCAAGTTGAGAGAGATAAATTGAACAAGTATG
GCCGTCCCCTATTGGGATGTACTATTAAACCTAAATTGGGGTTATCCGCTAAGAATTACGGTAGAGCTGT
TTATGAATGTCTACGTGGTGGACTTGACTTTACCAAAGATGATGAGAACTGTGAACTCCCAACCA
>35
AAATGTTGGATTTAAGGCTGGGGTTAAAGATTATAAATTAACTTATTATACTCCGGACTATGAAACCAAG
GATACCGATATTTTGGCAGCCTTTCGAGTCACTCCTCAACCCGGAGTTCCCCCGGAAGAAGCAGGGGC
CGCAGTAGCTGCCGAATCGTCTACTGGTACATGGACAACTGTGTGGACCGATGGGCTTACGAGCCTTGA
TCGTTACAAAGGGAGATGCTATCACATCGAGCCCGTTGCCGGAGAAGAAACTCAATTTATTGCTTATGT
AGCTTACCCATTAGACCTTTTTGAAGAAGGTTCTGTTACTAACATGTTTACTTCCATTGTGGGTAATGTAT
TTGGATTTAAAGCACTGCGTGCTCTACGTCTAGAAGATTTGCGAATCCCAACTGCGTATATTAAAACATT
TCAAGGCCCGCCTCACGGCATCCAAGTTGAGAGAGATAAATTGAACAAGTATGGTCGTCCCCTATTGG
GATGTACTATTAAACCAAAATTAGGGTTATCCGCTAAAAACTACGGCAGAGCAGTTTATGAATGTCTTC
GTGGTGGACTTGATTTTACTAAAGATGATGAGAACGTCAACTCCCAACCCTTTATGCGGTGGAGAGATC
GTTTCTTATTTTGTGCCGAAGCCATTTATAAAGCACAGGCTGAAACAGGTGAAATCAAAGGCCATTACT
TGAAT
>36
TAAAGCAAGCGTTGGATTTAAAGCTGGTGTTAAAGATTACAAATTGACTTATTATACTCCTGAGTATGAA
ACCCAAGATACTGATATCTTGGCAGCATTCCGAGTAAGTCCTCAACCGGGAGTTCCACCCGAAGAAGC
GGGGGCTGCAGTAGCTGCTGAATCTTCTACTGGTACATGGACAACTGTATGGACCGACGGACTTACCAG
TCTTGATCGTTACAAAGGACGATGCTACCACATCGAGCCTGTTGCTGGAGAAGAAAATCAATATATTTG
TTATGTAGCGTATCCCTTAGACCTTTTTGAAGAAGGTTCTGTTACTAACATGTTTACTTCCATTGTGGGTA
ACGTATTTGGGTTCAAAGCTCTGCGTGCTCTACGTTTGGAGGATTTACGAATCCCTGTTGCTTATATAAA
AACTTTCCAAGGCCCGCCTCACGGTATCCAAGTTGAGAGAGATAAATTGAACAAGTACGGCCGCCCCC
TATTGGGATGCACTATTAAACCAAAATTGGGGTTATCCGCTAAAAACTATGGTCGAGCATGTTATGAATG
TCTTCGTGGTGGACTTGATTTACCAAAGATGATGAAAACGTGAACTCCCAGCCGTT
>37

GAAACCAAAGATACTGATATCTTGGCAGCATTCAGAGTAACTCCTCAACCTGGAGTTCCGCCTGAGGA
AGCAGGGGCTGCAGTAGCTGCCGAATCTTCTACTGGTACCTGGACAACTGTGTGGACCGATGGGCTTAC
CAGCCTTGATCGTTATAAAGGAAGATGCTACCACATCGAGCCTGTTGCGGGAGAAGAGAATCAATATAT
ATGTTATGTAGCTTACCCCTTAGACCTTTTTGAAGAGGGTTCTGTTACTAATATGTTTACTTCCATTGTGGG
TAATGTATTTGGGTTCAAAGCCCTGCGTGCTCTACGTCTGGAGGATCTGAGAATCCCTACTGCATATGTT
AAAACTTTCCAAGGCCCGCCTCACGGCATCCAAGTTGAGAGAGATAAGTTGAACAAGTATGGCCGTCC
CCTATTGGGATGTACCATTAAACCGAAATTAGGTTTATCCGCTAAGAACTACGGTAGAGCTGTTTATGAA
TGTCTTCGTGGTGGACTTGATTTTACCAAGGATGATGAAAACGTCAACTCACAACCATTTATGCGTTGG
AGAGATCGTTTCTTATTTTGTGCCGAAGCAATTTATAAATCACAGGCTGAAACTGGTGAAATCAAAGGG
CATTACTTGAAT
>38
TAAAGCAGGTGTTGGATTCAAAGCTGGTGTTAAAGAGTACAAATTAACTTATTATACTCCTGAATACGA
AACCAAAGATACTGATATTTTGGCAGCATTCCGAGTAACTCCTCAACCGGGAGTTCCGCCGGAAGAAG
CGGGGGCCGCGGTAGCTGCCGAGTCTTCTACTGGTACATGGACAACTGTATGGACAGATGGACTTACC
AGCCTTGATCGTTACAAAGGGCGATGCTACCACATTGAGCCAGTTCCTGGAGAAGAAGATCAATTTATT
GCTTATGTAGCTTACCCCTTAGACCTTTTTGAAGAAGGTTCTGTTACTAACATGTTTACTTCCATTGTAGG
TAATGTATTTGGGTTCAAAGCCCTACGCGCCCTACGTTTGGAAGATTTGCGAATTCCCGTTGCTTATGTTA
AAACCTTCCAAGGACCGCCTCATGGGATTCAAGTCGAGAGAGATAAATTGAACAAGTATGGTCGTCCC
CTGTTGGGATGTACTATTAAACCAAAATTAGGTTTATCTGCTAAAAACTACGGTAGAGCGGTTTATGAAT
GTCTTCGTGGTGGACTTGATTTTACCAAAGATGATGAAAACGTGAACTCTCAACCATTTATGCGTTGGA
GAGACCGTTTCTTATTTTGTGCCGAAGCAATTTATAAATCGCAGGCTGAAACGGGTGAAATCAAAGGGC
ATTATTTGAAT
>39
TAAAGCAAGTGTTGGATTCAAAGCTGGTGTTAAAGATTATAAATTGACTTATTACACTCCTGAATATGAA
ACCAAAGATACTGATATCTTAGCAGCATTTCGAGTAACTCCTCAACCTGGAGTTCCCCCTGAAGAAGCA
GGGGCTGCAGTAGCTGCTGAATCTTCTACTGGTACATGGACAACTGTATGGACTGATGGGCTTACCAGC
CTTGATCGCTACAAAGGTCGCTGCTACCACATCGAGCCTGTTGCTGGAGAAGAAAATCAATTTATTGCT
TATGTAGCTTATCCCTTAGACCTTTTTGAAGAAGGTTCTGTTACTAACATGTTTACTTCCATTGTGGGTAA
TGTATTTGGGTTCAAGGCCCTGCGAGCTCTACGTCTGGAGGATTTGCGAATCCCCCCCGCATACACTAA
AACTTTCCAAGGCCCACCCCATGGCATCCAAGTTGAGAGAGATAAATTGAATAAGTATGGCCGCCCTCT
ATTGGGATGTACTATTAAACCTAAATTGGGACTATCCGCTAAGAATTATGGTAGAGCTGTTTATGAATGT
CTTCGCGGTGGACTTGATTTTACCAAAGATGATGAGAACGTGAATTCCCAACCATTTATGCGTTGGAGA
GACCGTTTCTTATTTTGTGCCGAAGCAATTTATAAATCACAGTCTGAAACCGGTGAAATCAAAGGACAT
TATTTGAAT
